# NAD^+^-dependent HDAC inhibitor stimulates *Monascus* pigment production but inhibit citrinin

**DOI:** 10.1186/s13568-017-0467-1

**Published:** 2017-08-23

**Authors:** Yan Hu, Youxiang Zhou, Zejing Mao, Huihui Li, Fusheng Chen, Yanchun Shao

**Affiliations:** 1 College of Food Science and Technology, Huazhong Agricultural University, Wuhan, 430070 People’s Republic of China; 2 Institute of Quality Standard and Testing Technology for Agro-Products, Hubei Academy of Agricultural Science, Wuhan, 430064 People’s Republic of China; 3 College of Cuisine and Food Technology, Wuhan Business University, Wuhan, 430056 People’s Republic of China

**Keywords:** *Monascus ruber*, Epigenetic modifier, Dihydrocoumarin, Secondary metabolites, Organism growth

## Abstract

*Monascus* species are edible fungi due to the production of food colorant and other beneficial compounds. Hence, it has been an attractive thesis to improve their productivities. Increasing numbers of investigations revealed that regulating the activities of histone deacetylases can significantly perturb secondary metabolites (SM) production at a global level. In this study, dihydrocoumarin (DHC, an inhibitor of the Sirtuin family of NAD^+^-dependent deacetylases) was used to treat *Monascus ruber* for evaluating its effects on organism growth and SM production. The results revealed that the variation trends of colonial sizes, biomass and mycotoxin were in a dose-dependent manner. Generally, they decreased with the increased DHC concentrations in the designed range. But the variation trend of pigment was different. Comparison of SM profile, three new peaks occurred to the mycelia extractions from DHC-treated strain corresponding to molecular weights 402, 416 and 444, respectively. These three compounds were identified as Monasfluol B, Monascus azaphilone C and acetyl-monasfluol B (a new *Monascus* chemical pigment structure). In short, DHC can stimulate *M. ruber* strain to produce more pigment-like polyketides but inhibition of mycotoxin (citrinin).

**Electronic supplementary material:**

The online version of this article (doi:10.1186/s13568-017-0467-1) contains supplementary material, which is available to authorized users.

## Introduction

Filamentous fungi are important sources of secondary metabolites (SM) which can produce antibiotics, organic acid, immunosuppressants, siderophores, antitumor agents and toxins (Brakhage 2013). *Monascus* species are very important microbial resources in oriental countries due to their abilities to produce food additives and pharmaceutical ingredients, such as edible pigments, natural antioxidant dimerumic acid, anti-hyper-cholesterolemic agent monacolins, hypotensive agent gamma-amino butyric acid (GABA) as well as sterol and isoflavones. Hence, it has been an attractive thesis to improve their productivities. Bioinformatics analysis revealed that the genome of *Monascus* strains contained more gene backbones encoding SMs than currently characterized compounds (Chen et al. 2015), suggesting *Monascus* strains have great potential to produce new compounds.

It is well-known that SM production is a complicated process, which is not only linked to the expression of special gene clusters, but also with multiply modulatory strategies (Bonasio et al. 2010). The increasing numbers of investigations revealed that the expression level of SM gene clusters has been linked to epigenetic mechanisms, which are functionally relevant changes to the genome that do not involve in a change in the nucleotide sequence (Fuchs and Quasem 2014). Histone modification is one of the examples of these mechanisms leading to variation of SM production. Particularly, acetylation, balanced by the opposing actions of histone acetyltransferases (HATs) and histone deacetylases (HDACs), serves as a key regulatory mechanism for chromatin structure and gene expression (Brakhage and Schroeckh 2011; Bulger 2005). Generally, histone acetylation links to the transcription activation (Albright et al. 2015; Robyr et al. 2002). Several research groups have demonstrated that it is a promising approach to get new compounds by suppressing the activity of HDACs with epigenetic modifiers (Asai et al. 2012a, b; Cichewicz 2010; Mao et al. 2015; Matthew et al. 2016; Yang et al. 2013 **)**. For examples, *Aspergillus niger* was treated with suberoylanilide hydroxamic acid leading to the isolation of nigerone A (Henrikson et al. 2009). Similarly, new compound named chlorinated polyketide was obtained from a *Daldinia* sp. (Du et al. 2014).

In recent years, several approaches, such as mutation breeding (Chen and Hu 2005), protoplast fusion (Klinsupa et al. 2016) and genetic modifications (Lee et al. 2013; Xu et al. 2009) were explored to increase SM yields in *Monascus* spp., but no epigenetic modifiers were tested to treat *Monascus* spp. Dihydrocoumarin (DHC) is a broad-acting inhibitor of the Sirtuin family of NAD^+^-dependent deacetylases participating in a wide variety of physiological and biochemical processes, which is also approved as a food-grade flavor agent (Jacobi et al. 2016). In current study, *M. ruber* was incubated in the presence or absence of DHC. Then, its growth, spore development, production of pigments and mycotoxin as well as SM profile were analysed. At the range of tested concentration of DHC, pigment yields increased but citrinin and growth speed decreased. Combination of multiple analytical techniques identified a new *Monascus* chemical pigment structure named 1-(6a*RS*,9a*RS*)-9-hexanoyl-6a-methyl-6,8-dioxo-6a,8,9,9a-tetrahydro-6*H*-furo[2,3-h]isochromen-3-)propanyl-2acetate. This study supplied an alternative approach to increase the production of *Monascus* pigment-like polyketides but reduce mycotoxin (citrinin).

## Materials and methods

### Strains and culture conditions

*Monascus ruber* M7 was cultivated on potato dextrose agar (PDA) slants at 28 ^°^C for 7 days for collecting fresh spores to do following measurements.

### Measurement of colony growth and spore development as well as biomass

5 µL of fresh spore (10^4^/mL) was spotted on PDA plate with or without DHC and cultivated on 28 °C. Colony diameter was measured at the 10th day. The conidia and cleistothecia were observed using an Olympus BH2 compound microscope with differential interference contrast optics.

For biomass measurement, 1 mL of fresh 10^6^/mL spores was seeded into 50 mL of PDB containing different concentration (0, 0.5, 1.0, 2.0, 5.0, 10 mM) of DHC in 250 mL Erlenmeyer flasks with continuous shaking at 150 rpm at 28 °C for 10 days (three replicates per concentration). Then fermented broths were centrifuged for 5 min at 2200*g*. The precipitation was washed three times with distilled water and dried to a constant weight at 45 °C as biomass, and the supernatant was collected to detect mycotoxin (citrinin).

### Detection of pigments and mycotoxin (citrinin)

Pigments production was measured according to the approach described by Lai et al. (2011) with minor modification. Briefly, about 0.1 g dry biomass was extracted with 10 mL of 70% ethanol at room temperature for 2 h. Then mycelia extraction was scanned by UV–Vis Spectrophotometer (UV-1700, Shimadzu, Japan) from 300 nm to 600 nm using 70% ethanol as the negative control. The result was expressed as optical density units (multiply the absorbance values by the dilution factors) per gram.

For the determination of citrinin, after the filtration described above, 10 mL of the filtrate was concentrated by a rotary vacuum evaporator and then the pellet was dissolved in 1 mL of methanol, which was subjected to HPLC analysis following previously described method (Yang et al. 2012). A citrinin standard compound (Sigma, USA) was used to confirm the HPLC analysis.

### UPLC-PDA analysis of secondary metabolites

0.1 g of lyophilized mycelia was ground into powder with liquid nitrogen, and submerged in 2 mL of methanol for 2 h, which was centrifuged at 10,000 rpm for 5 min. The supernatant was filtered by 0.22 μm hydrophilic Nylon membrane and subjected to ultra performance liquid chromatography with photodiode array detector (UPLC-PDA) analysis. The optimized condition was as follows: 2 μL of sample was separated on the Acquity BEH C18 column (1.7 µm, 2.1 mm × 100 mm, Waters, USA), at 30 ^°^C with a linear gradient starting with 35% (v/v) acetonitrile (ACN)/water to 90% ACN in 22 min and detected at 360 nm, the flow rate was set at 0.3 mL/min.

### Recovery and identification of compounds with new peaks

10 µL of the extracted sample above was spotted on TLC plate coated with Silica gel G, and developed with methylbenzene:ethyl acetate:formic acid = 7:3:1 (v:v) as mobile phase. Those bands with significant augment were recovered to solve in methanol and filtered through 0.22 µm hydrophilic nylon membrane. The supernatant was separated on the semi-preparation Inertsil ODS-3 column (10 mm × 250 mm, 5 μm, Shimadzu, Japan), equipped with a C18 guard column (4.6 mm × 12.5 mm, 5 μm, Phenomenex) at 30 °C with a flow rate of 3 mL/min. Separation was performed using isocratic elution (acetonitrile/water) with a solvent ratio of 80:20 at 360 nm. The purified compounds were stored at 4 °C for structure analysis.

The mass analysis was performed in an Acquity UPLC™-TQD system (Waters, Milford, MA, USA), using an Acquity UPLC BEH C18 column (100 mm × 2.1 mm, 1.7 μm, from Waters). MS detection was performed using an Acquity TQD tandem quadrupole mass spectrometer (Waters, Manchester, UK). The instrument was operated with an electrospray (ESI) source in positive ion scan mode. The ionization source parameters in positive mode were capillary voltage 2.5 kV, extractor voltage 4 V, source temperature 150 °C, desolvation temperature 350 °C, cone gas flow 60 L/h, and desolvation gas flow 600 L/h (both gases were nitrogen).

^1^H-NMR, ^13^C-NMR and 2D spectra (COSY, HSQC, HMBC) of those new compounds were acquired using AV400 NMR (Bruker, Germany). NMR spectra were referenced to the signals of the solvent, methanol I-d4.

### Real-time quantitative PCR analysis

The expression levels of genes encoding pigment and citrinin of *M. ruber* M7 were analyzed by a real-time quantitative PCR analysis (RT-qPCR). 1 mL of fresh 10^5^/mL spores was seeded into 50 mL of PDB containing 2 mM of DHC and incubated at 28 °C with continuous shaking at 150 rpm, and samples were taken at the 5th day and the 11th day, respectively. RT-qPCR was performed according to the method described by Liu et al. (2016). GAPDH was used as a reference gene. The primers used in this study are listed in Table 1.

**Table 1:** Primers used for real-time quantitative PCR

Primers	Sequence (5′–3′)	Fragment length (bp)
MpksCT-F	TTTTCAGCGTCGTGGAATAGC	294
MpksCT-R	TAACATCTTCGTCGCATCAGC	
MpigA-F	GTCATTGGCATGTCGTGTAAGG	182
MpigA-R	GCATCGTGGTCTCGGATAAAG	
GAPDH-F	CAAGCTCACTGGCATGTCTATG	243
GAPDH-R	AAGTTCGAGTTGAGGGCGATA	

*F* forward primer, *R* reverse primer

## Results

### Effects of DHC on colony growth and spore development

**Fig. 1:**
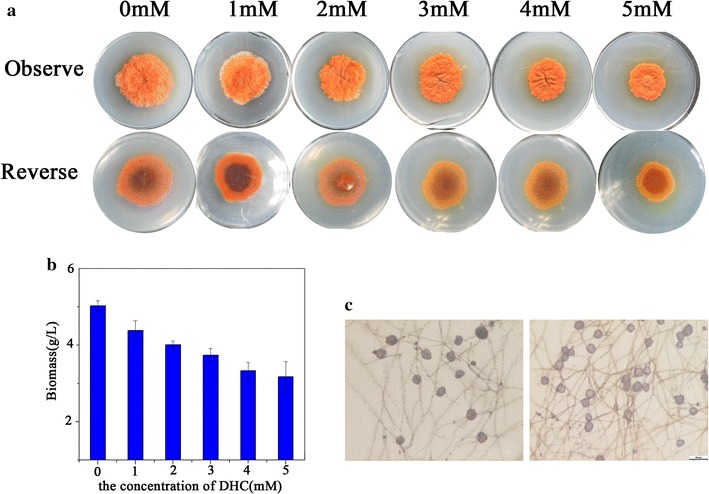
Comparison of the *Monascus ruber* M7 strain in colony growth, biomass and cleistothecia development in the presence or absence of DHC. **a** Colony growth at different dose of DHC growing on PDA at 28 °C for 10 days. **b** The biomass (dry cell weight) at different dose of DHC growing in PDB at 28 °C for 10 days with continuous shaking at 150 rpm. **c** Cleistothecia growing on PDA at 28 °C for 10 days. Error bars indicate standard deviation

Before detecting the variation of SMs due to DHC treatment, the colony growth on PDA and biomass in PDB were evaluated. The results showed that both colony growth and dry biomass weight were dependent on DHC concentration. With the increasing concentration of DHC, the colony size and biomass were reduced, suggesting DHC can inhibit the growth of *M. ruber* in tested concentration (Fig. 1a, b). Comparatively, the amount of cleistothecia from DHC-treated strain was more than that of original strain in equal observed area (Fig. 1c).

### Effects of DHC on the production of pigment and citrinin

**Fig. 2:**
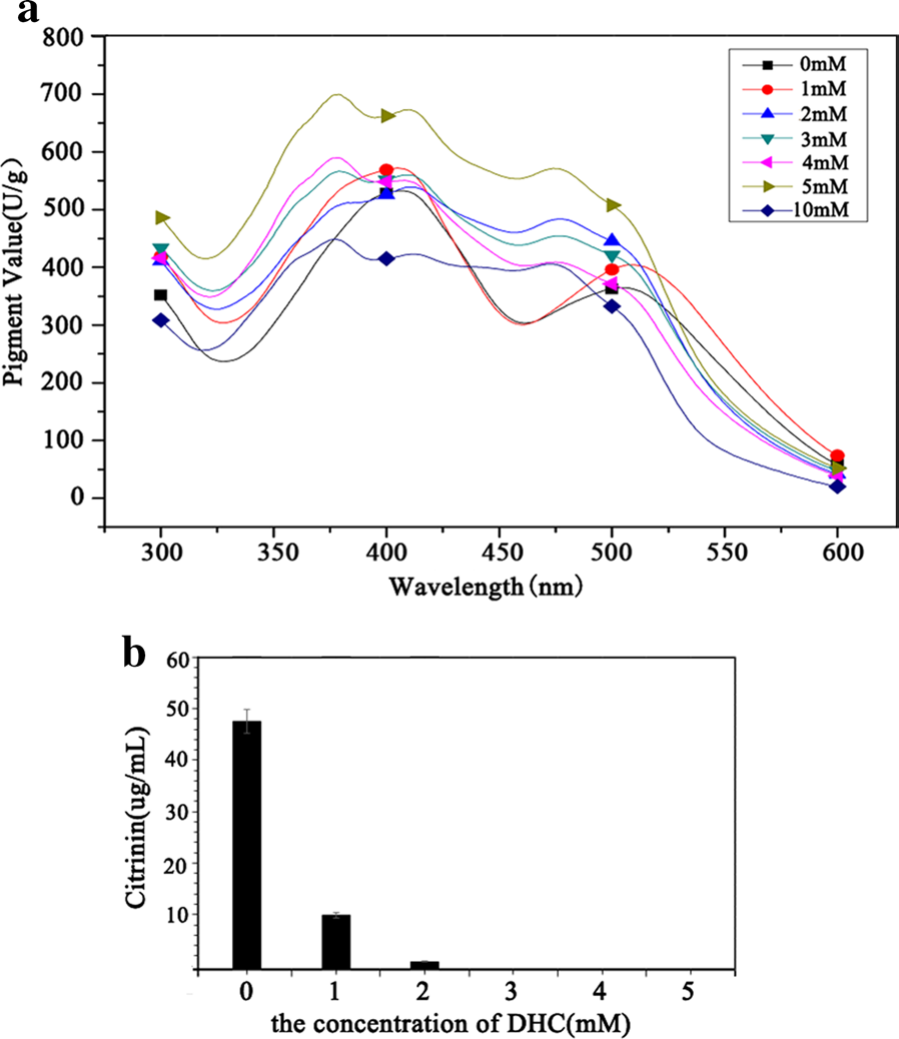
The effect of DHC on intracellular pigment and extracellular citrinin. **a** Pigment production at different dose of DHC at the 10th day. **b** The concentration of citrinin at different dose of DHC at the 10th day

Relative quantities of intracellular pigment growing in PDB were measured using UV–Vis spectroscopy. For original strain, two absorbance peaks, around 390 and 510 nm, were observed. Comparatively, blue shift occurred to the absorbance peaks from the mycelia extraction of DHC-treated strain except the 1 mM of DHC. Furthermore, pigment production improved with the increasing doses of DHC ranged from 1 to 5 mM, but 10 mM of DHC can inhibit pigment production (Fig. 2a). The citrinin yield secreted in PDB was measured using HPLC. Interestingly, the result revealed that citrinin content was decreased with increased dose of DHC, suggesting that DHC can inhibit citrinin production. When DHC concentration amounted to 3 mM in the PDB, citrinin can’t be detected via the method described in current study because its content was lower than the detection limit (citrinin concentration <10 ng/mL) (Fig. 2b).

### Effects of DHC on secondary metabolic profile

**Fig. 3:**
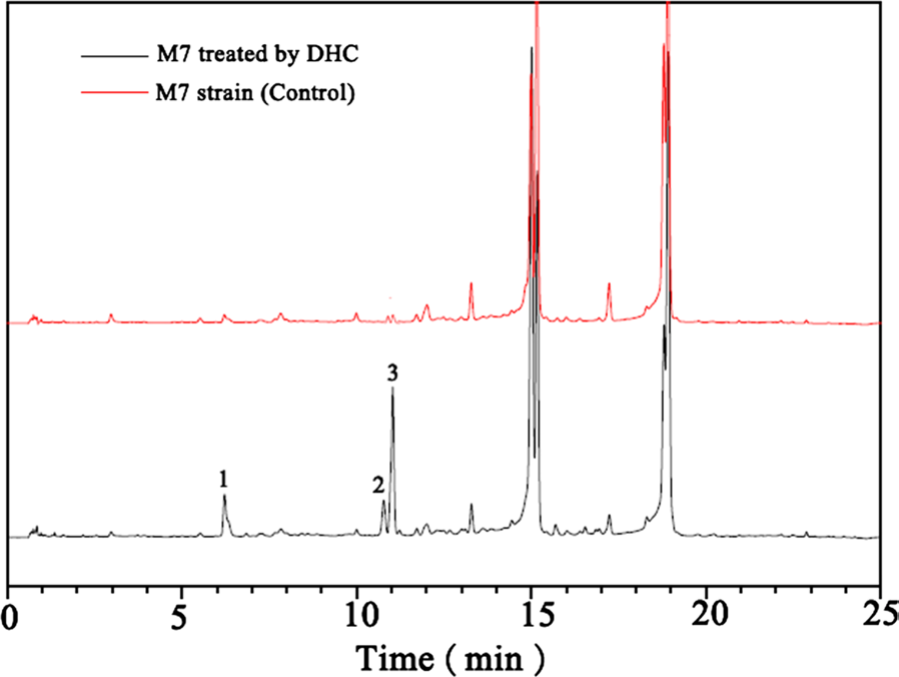
UPLC-PDA profiles at 360 nm of the methanol extractions from *M. ruber* M7 growing in the presence or absence of DHC

A great number of investigations demonstrated that exposure of fungi to epigenetic modifiers can enhance the production of SMs. Considering this, mycelia extraction from DHC-treated strain was subjected to UPLC-PDA to discover new SM(s). Among the tested DHC concentration, 2 mM of DHC can significantly change SM profile. The results suggested that three new peaks occurred to the mycelia extractions from DHC-treated strain, corresponding to the retention time at 6.215, 10.773 and 11.033 min, respectively (Fig. 3).

**Fig. 4:**
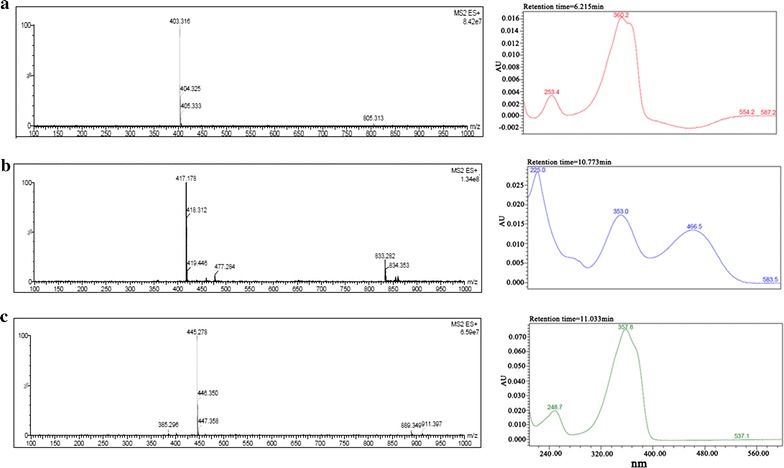
Mass spectra and spectrogram of compounds 1, 2 and 3 detected by UPLC-MS. **a** Compound 1. **b** Compound 2. **c** Compound 3

**Fig. 5:**
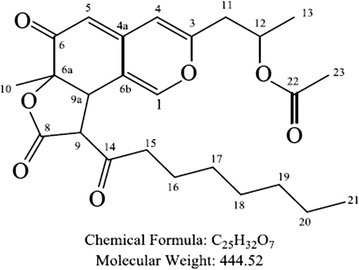
The chemical structure of compound 3

**Fig. 6:**
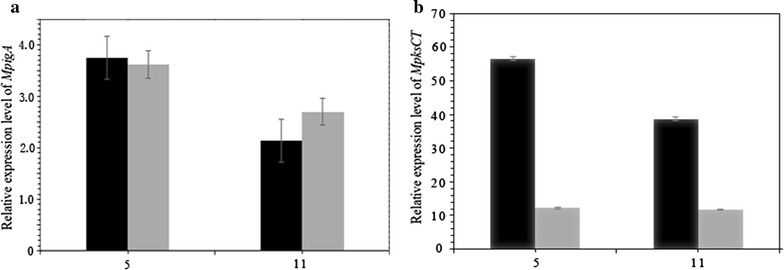
The transcription level of polyketide synthases genes encoding pigment and citrinin. **a** *MpigA* gene encoding pigment synthesis. **b** *MpksCT* gene encoding citrinin synthesis

After that, the extraction was spotted on TLC plate for collecting the significantly different bands. UPLC-MS identified their molecular weights as 402 (compound 1), 416 (compound 2) and 444 (compound 3) (Fig. 4). Combining those spectra of isolated compounds and the published documents (Wu et al., 2013; Balakrishnan et al. 2014), compound 1 and 2 were identified as Monasfluol B and Monascus azaphilones C (Figs. 4a, b; 5, Additional file 1: Figure S1; Tables 2, 3). Combination of ^1^H-NMR, ^13^C-NMR and 2D spectra including COSY, HSQC and HMBC identified compound 3 as acetyl-monasfluol B (Fig. 4c; Additional file 1: Figure S2; Table 4). Among of them, acetyl-monasfluol B is a new polyketide-like structure named 1-(6a*RS*,9a*RS*)-9-hexanoyl-6a-methyl-6,8-dioxo-6a,8,9,9a-tetrahydro-6*H*-furo[2,3-h] isochromen-3-)propanyl-2acetate (Fig. 6).

**Table 2:** ^1^H and ^13^C NMR data for compound 1 (400 MHz in methanol I-d4 δ ppm)

No.	Position	δ_C_	δ_H_ (mult, J in Hz)
1	1	148.4	7.56 (s, 1H)
2	3	163.0	
3	4	105.6	6.34 (s, 1H)
4	4a	149.9	
5	5	109.9	5.39 (d, *J* = 1.2, 1H)
6	6	194.1	4.08 (m, 1H)
7	6a	84.1	1.31 (d, *J* = 8.2, 3H)
8	6b	116.3	
9	8	170.1	
10	9	66.3	3.97 (s, 1H)
11	9a	43.9	3.04 (br s, 1H)
12	10	23.5	1.54 (s, 3H)
13	11	43.7	2.56 (ddd, *J*_*1*_ = 4.5, J2 = 8.5, J3 = 14.2, 2H)
14	12	56.9	4.08 (m, 1H)
15	13	23.7	1.31 (d, J = 8.2, 3H)
16	14	203.8	
17	15	43.4	3.32 (dt, *J*_*1*_ = 1.6, J2 = 3,3, 2H)
18	16	23.5	1.60 (d, *J* = 7.7, 2H)
19	17	30.1	1.24 (o, 2H)
20	18	31.5	1.24 (o, 2H)
21	19	32.3	1.24 (o, 2H)
22	20	23.5	1.24 (o, 2H)
23	21	14.3	0.91 (t, *J* = 7.1, 3H)

**Table 3:** ^1^H and ^13^C NMR data for compound 2 (400 MHz in methanol I-d_4_ δ ppm)

No.	Position	δ_C_	δ_H_ (mult, J in Hz)
1	C-1	149.76	7.49 (s, 1H)
2	C-3	161.40	
3	C-4	110.07	6.30 (s, 1H)
4	C-4a	147.93	
5	C-5	106.07	5.36 (s, 1H)
6	C-6	194.21	
7	C-6a	84.12	
8	C-6b	116.33	
9	C-8	171.06	
10	C-9	57.11	3.92 (s, 1H)
11	C-9a	43.64	3.28 (s, 1H)
12	C-10	23.46	1.50 (s, 3H)
13	C-11	40.55	2.68 (d, *J* = 6.4, 2H)
14	C-12	69.39	5.36 (dd, *J* = 6.3, 12.7, 1H)
15	C-13	20.05	1.27 (s, 3H)
16	C-14	203.86	
17	C-15	43.98	2.94 (dt, *J* = 7.3, 7.3, 18.3, 2H)
18	C-16	23.47	1.56 (m, 2H)
19	C-17	32.28	1.26 (m, 2H)
20	C-18	23.71	1.25 (m, 2H)
21	C-19	14.26	0.86 (t, *J* = 7.1, 3H)
22	C20	172.04	
23	C-21	14.26	1.95 (s, 3H)

**Table 4:** ^1^H and ^13^C NMR data for compound 3 (400 MHz in methanol I-d_4_ δ ppm)

No.	Position	δ_C_	δ_H_ (mult, J in Hz)
1	C-1	149.77	7.46 (s, 1H)
2	C-3	161.38	
3	C-4	110.07	6.27 (s, 1H)
4	C-4a	147.93	
5	C-5	106.07	5.33 (s, 1H)
6	C-6	194.20	
7	C-6a	84.08	
8	C-6b	116.34	
9	C-8	171.03	
10	C-9	58.67	3.28 (s, 1H)
11	C-9a	43.65	3.89 (s, 1H)
12	C-10	23.68	1.51 (s, 3H)
13	C-11	40.55	2.65 (d, J = 6.3, 2H)
14	C-12	69.39	5.16 (m, 1H)
15	C-13	20.05	1.26 (d, J = 6.4, 3H)
16	C-14	203.86	
17	C-15	43.93	2.58 (dt, J_1_ = 7.3, J_2_ = 18.3, 1H)
18	C-16	23.47	1.51 (m, 2H)
19	C-17	30.21	1.26 (o, 2H)
20	C-18	30.04	1.26 (o, 2H)
21	C-19	32.88	1.26 (o, 2H)
22	C-20	24.03	1.26 (o, 2H)
23	C-21	14.44	0.9 (t, J = 7.1, 3H)
24	C-22	171.97	
25	C-23	21.02	1.99 (s, 3H)

### Effects of DHC on gene expression responsible for the synthesis of pigment and citrinin

In *M. ruber*, genes involving in synthesis of pigment and citrinin have been identified (He and Cox 2016; Chen et al. 2017). To understand the effects of exposure to DHC on the production of pigment and citrinin at the molecular level, we analyzed the expression level of *MpigA* and *MpksCT* encoding polyketide synthases responsible for the synthesis of pigment and citrinin, respectively. As shown in Fig. 6, the transcript level of *MpigA* was up-regulated, but that of *MpksCT* was significantly down-regulated in the presence of 2 mM of DHC at the tested time, which is in accordance with the production of pigment and citrinin.

## Discussion

In recent years, researchers adopted diverse approaches to improve bioactive compounds or discover new compounds for industrial application. A series of investigations proved that acetylation modification plays an important role in regulating SM production via modification of HDACs activities (Albright et al. 2015). In this study, DHC has multiple effects on SM production of *M. ruber*. Firstly, suitable range of DHC can improve pigment yields but reduce mycotoxin (citrinin). More importantly, new pigment-like polyketide was discovered. So DHC is an alternative agent to stimulate *M. ruber* to produce much more pigment-like compounds.

Acetylation modification not only regulates SMs production, also involve in other bioprocesses such as fungi growth, spore development, metabolism and stress response (Niu et al. 2015). Addition of DHC can inhibit colony growth and biomass probably attributed to weakened activity of NAD^+^-dependent HDACs. During the growing period of *M. ruber*, glucose is the single carbon for normal growth, but inhibition of HDACs activity probably affected the glucose metabolism to retard colony growth. It has been reported that Sirtuin proteins regulated gene expression related to glucose metabolism in yeast and mammals, suggesting that Sirtuin-like HDACs are very conserved in biological function (Kang et al. 2017; Kugel and Mostoslavsky 2014). Inhibition or deletion of HDACs is a feasible approach to discover new compounds by activating unknown SM cluster or modifying non-histone protein. In this study, the new identified compound (acetyl-Monasfluol B) is the derivative of a known pigment Monasfluol B, the hydroxyl group linked to C-12 of which was replaced by acetyl group. Since acetyl-Monasfluol B was not a compound from unknown SM cluster, it was possible that acetylation modification occurred to catalytic enzyme involving in pigment conversion.

It is a big issue that the correlation between fungal development and secondary metabolism. In current study, the variation of the cleistothecia and the production of SMs such as pigment and citrinin may be a logical strategy to adapt to their changing environment (Etxebeste et al. 2010). In *Aspergillus oryzae* RIB40, HstD (a homolog of Sirtuin) coordinates secondary metabolism and development through control of the global regulator LaeA, suggesting that member of signaling pathway connects acetylation modification with its modulation processes (Kawauchi et al. 2013). In conclusion, DHC can be used to raise pigment yield and its derivative but inhibit mycotoxin, laying the foundation for further exploring new metabolites.

## Additional file

**Additional file 1: Figure S1.** NMR spectra for compound 2. (A) H-NMR. (B) C-NMR. (C) COSY. (D) HMBC. (E) HSQC. (F)TOCSY. **Figure S2**. NMR spectra for compound 3. (A) H-NMR. (B) C-NMR. (C) COSY. (D) HMBC. (E) HSQC. (F)TOCSY.

## Abbreviations

DHC: dihydrocoumarin
NAD: nicotinamide adenine dinucleotide
SM: secondary metabolite
GABA: gamma-amino butyric acid
HATs: histone acetyltransferases
HDACs: histone deacetylases
PDA: potato dextrose agar
UPLC-PDA: ultra performance liquid chromatography-photodiode array detector
ACN: acetonitrile
TLC: thin-layer chromatography
ESI: electrospray
NMR: nuclear magnetic resonance
COSY: correlation spectroscopy
HSQC: heteronuclear single quantum coherence spectroscopy
HMBC: heteronuclear multiple bond correlation
RT-qPCR: real-time quantitative PCR analysis
GAPDH: glyceraldehydes 3-phosphate dehydrogenase
PDB: potato dextrose broth

## References

[CR1] Albright JC, Henke MT, Soukup AA, McClure RA, Thomson RJ, Keller NP, Kelleher NL (2015). Large-scale metabolomics reveals a complex response of *Aspergillus nidulans* to epigenetic perturbation. ACS Chem Biol.

[CR2] Asai T, Chung YM, Sakurai H, Ozeki T, Chang FR, Wu YC, Yamashita K, Oshima Y (2012). Highly oxidized ergosterols and isariotin analogs from an entomopathogenic fungus, *Gibellula formosana*, cultivated in the presence of epigenetic modifying agents. Tetrahedron.

[CR3] Asai T, Chung YM, Sakurai H, Ozeki T, Chang FR, Yamashita K, Oshima Y (2012). Aromatic polyketide production in *Cordyceps indigotica*, an entomopathogenic fungus, induced by exposure to a histone deacetylase inhibitor. Org Lett.

[CR4] Balakrishnan B, Chen CC, Pan TM, Kwon HJ (2014). Mpp7 controls regioselective Knoevenagel condensation during the biosynthesis of *Monascus* azaphilone pigments. Tetrahedron Lett.

[CR5] Bonasio R, Tu S, Reinberg D (2010). Molecular signals of epigenetic states. Science.

[CR6] Brakhage AA (2013). Regulation of fungal secondary metabolism. Nat Rev Microbiol.

[CR7] Brakhage AA, Schroeckh V (2011). Fungal secondary metabolites—strategies to activate silent gene clusters. Fungal Genet Biol.

[CR8] Bulger M (2005). Hyperacetylated chromatin domains: lessons from heterochromatin. J Biol Chem.

[CR9] Chen FS, Hu XQ (2005). Study on red fermented rice with high concentration of monacolin K and low concentration of citrinin. Int J Food Microbiol.

[CR10] Chen WP, He Y, Zhou YX, Shao YC, Feng YL, Li M, Chen FS (2015). Edible filamentous fungi from the species *Monascus*: early traditional fermentations, modern molecular biology, and future genomics. Compr Rev Food Sci.

[CR11] Chen WP, Chen RF, Liu QP, He Y, He K, Ding XL, Kang LJ, Guo XX, Xie NN, Zhou YX, Lu YY, Cox RJ, Molnar I, Li M, Shao YC, Chen FS (2017). Orange, red, yellow: biosynthesis of azaphilone pigments in *Monascus* fungi. Chem Sci.

[CR12] Cichewicz RH (2010). Epigenome manipulation as a pathway to new natural product scaffolds and their congeners. Nat Prod Rep.

[CR13] Du L, King JB, Cichewicz RH (2014). Chlorinated polyketide obtained from a *Daldinia* sp. treated with the epigenetic modifier suberoylanilide hydroxamic acid. J Nat Prod.

[CR14] Etxebeste O, Garzia A, Espeso EA, Ugalde U (2010). *Aspergillus nidulans* asexual development: making the mostof cellular modules. Trends Microbiol.

[CR15] Fuchs SM, Quasem I (2014). Budding yeast as a model to study epigentics. Drug Discov Today Dis Models.

[CR16] He Y, Cox RJ (2016). The molecular steps ofcitrinin biosynthesis in fungi. Chem Sci.

[CR17] Henrikson JC, Hoover AR, Joyner PM, Cichewicz RH (2009). A chemical epigenetics approach for engineering the in situ biosynthesis of a cryptic natural product from *Aspergillus niger*. Org Biomol Chem.

[CR18] Jacobi JL, Yang B, Li X, Menze AK, Laurentz SM, Janle EM, Ferruzzi MG, McCabe GP, Chapple C, Kirchmaier AL (2016). Impacts on sirtuin function and bioavailability of the dietary bioactive compound dihydrocoumarin. Plos ONE.

[CR19] Kang WK, Devare M, Kim JY (2017). HST1 increases replicative lifespan of a sir2Δ mutant in the absence of PDE2 in *Saccharomyces cerevisiae*. J Microbiol.

[CR20] Kawauchi M, Nishiura M, Iwashita K (2013). Fungus-specific sirtuin HstD coordinates secondary metabolism and development through control of LaeA. Eukaryot Cell.

[CR21] Klinsupa W, Phansiri S, Thongpradis P, Yongsmith B, Pothiratana C (2016). Enhancement of yellow pigment production by intraspecific protoplast fusion of *Monascus* spp. yellow mutant (ade(−)) and white mutant (prototroph). J Biotechnol.

[CR22] Kugel S, Mostoslavsky R (2014). Chromatin and beyond: the multitasking roles for SIRT6. Trends Biochem Sci.

[CR23] Lai Y, Wang L, Qing L, Chen FS (2011). Effects of cyclic AMP on development and secondary metabolites of *Monascus ruber* M-7. Lett Appl Microbiol.

[CR24] Lee SS, Lee JH, Lee I (2013). Strain improvement by overexpression of the *laeA* gene in *Monascus pilosus* for the production of *monascus*-fermented rice. J Microbiol Biotechnol.

[CR25] Liu QP, Cai L, Shao YC, Zhou YX, Li M, Wang XH, Chen FS (2016). Inactivation of the global regulator LaeA in *Monascus ruber* results in a species-dependent response in sporulation and secondary metabolism. Fungal Biol.

[CR26] Mao XM, Xu W, Li D, Yin WB, Chooi YH, Li YQ, Tang Y, Hu Y (2015). Epigenetic genome mining of an endophytic fungus leads to the pleiotropic biosynthesis of natural products. Angew Chem Int Ed.

[CR27] Matthew TH, Soukup AA, Goering AW, McClure RA, Thomson RJ, Keller NP, Kelleher NL (2016). New Aspercryptins, lipopeptide natural products, revealed by HDAC inhibition in *Aspergillus nidulans*. ACS Chem Biol.

[CR28] Niu XL, Hao XR, Hong ZY, Chen LF, Yu X, Zhu XD (2015). A putative histone deacetylase modulates the biosynthesis of pestalotiollide B and conidiation in *Pestalotiopsis microspore*. J Microbiol Biotechnol.

[CR29] Robyr D, Suka Y, Xenarios I, Kurdistani SK, Wang A, Suka N, Grunstein M (2002). Microarray deacetylation maps determine genome-wide functions for yeast histone deacetylases. Cell.

[CR30] Wu MD, Cheng MJ, Yech YJ, Chen YL, Chen KP, Yang PH, Chen IS, Yuan GF (2013). Monascus azaphilones A–C, three new azaphilone analogues isolated from the fungus Monascus purpureus BCRC 38108. Nat Prod Res.

[CR31] Xu MJ, Yang ZL, Liang ZZ, Zhou SN (2009). Construction of a *Monascus purpureus* mutant showing lower citrinin and higher pigment production by replacement of *ctnA* with *pks1* without using vector and resistance gene. J Agric Food Chem.

[CR32] Yang YS, Li L, Li X, Shao YC, Chen FS (2012). *mrflbA*, encoding a putative FlbA, is involved in aerial hyphal development and secondary metabolite production in *Monascus ruber* M-7. Fungal Biol.

[CR33] Yang XL, Awakawa T, Wakimoto T, Abe I (2013). Induced production of novel prenyldepside and coumarins in endophytic fungi *Pestalotiopsis acaciae*. Tetrahedron Lett.

