# Supplementary material for: NAD+-dependent HDAC inhibitor stimulates Monascus pigment production but inhibit citrinin
Source: AMB Express. 2017 Aug 23;7:166. doi: 10.1186/s13568-017-0467-1 (PMC5568183; doi:10.1186/s13568-017-0467-1)

Journal Name: Applied Microbiology and Biotechnology

Manuscript Title:

NAD^+-dependent HDAC inhibitor stimulates *Monascus* pigment production but inhibit citrinin

Authors:

Yan Hu[1£], Youxiang Zhou[2£], Zejing Mao[1], Huihui Li[3], Fusheng Chen[1], Yanchun Shao[1*]

[£]: Yan Hu and Youxiang Zhou contributed equally to the manuscript.

1: College of Food Science and Technology, Huazhong Agricultural University, Wuhan, 430070 P.R. China

2: Institute of Quality Standard and Testing Technology for Agro-Products, Hubei Academy of Agricultural Science, Wuhan, 430064 P.R. China

3: College of Cuisine and Food Technology, Wuhan Business University, Wuhan, 430056 P.R. China

Correspondence: Yanchun Shao

Affiliation: Huazhong Agricultural University, Wuhan, 430070 P.R. China

Email: yanchunshao@mail.hzau.edu.cn

Tel: 86-27-87282111

Fax: +86-27-87288373

ELECTRONIC SUPPLEMENTARY INFORMATION

Figure S1 NMR spectra for compound 2. (A) H-NMR. (B) C-NMR. (C) COSY. (D) HMBC. (E) HSQC. (F)TOCSY.

Figure S2 NMR spectra for compound 3. (A) H-NMR. (B) C-NMR. (C) COSY. (D) HMBC. (E) HSQC. (F)TOCSY.

(A)

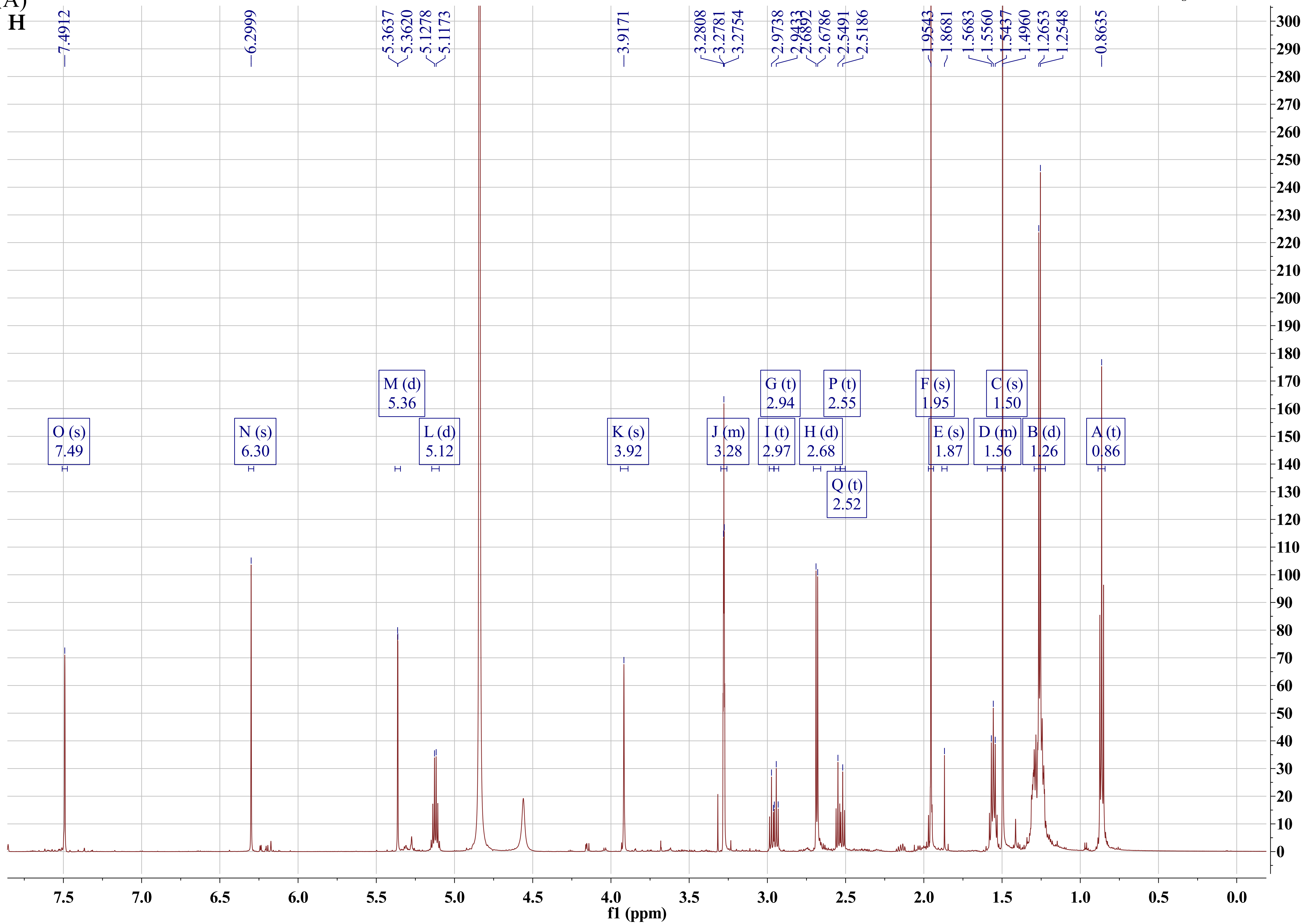
H
7.4912
6.2999
5.3637
5.3620
5.1278
5.1173
3.9171
3.2808
3.2781
3.2754
2.9738
2.9433
2.6892
2.6786
2.5491
2.5186
1.9543
1.8681
1.5683
1.5560
1.5437
1.4960
1.2653
1.2548
0.8635
O (s) 7.49
N (s) 6.30
M (d) 5.36
L (d) 5.12
K (s) 3.92
J (m) 3.28
G (t) 2.94
I (t) 2.97
H (d) 2.68
P (t) 2.55
Q (t) 2.52
F (s) 1.95
E (s) 1.87
D (m) 1.56
C (s) 1.50
B (d) 1.26
A (t) 0.86
f1 (ppm)

(B)

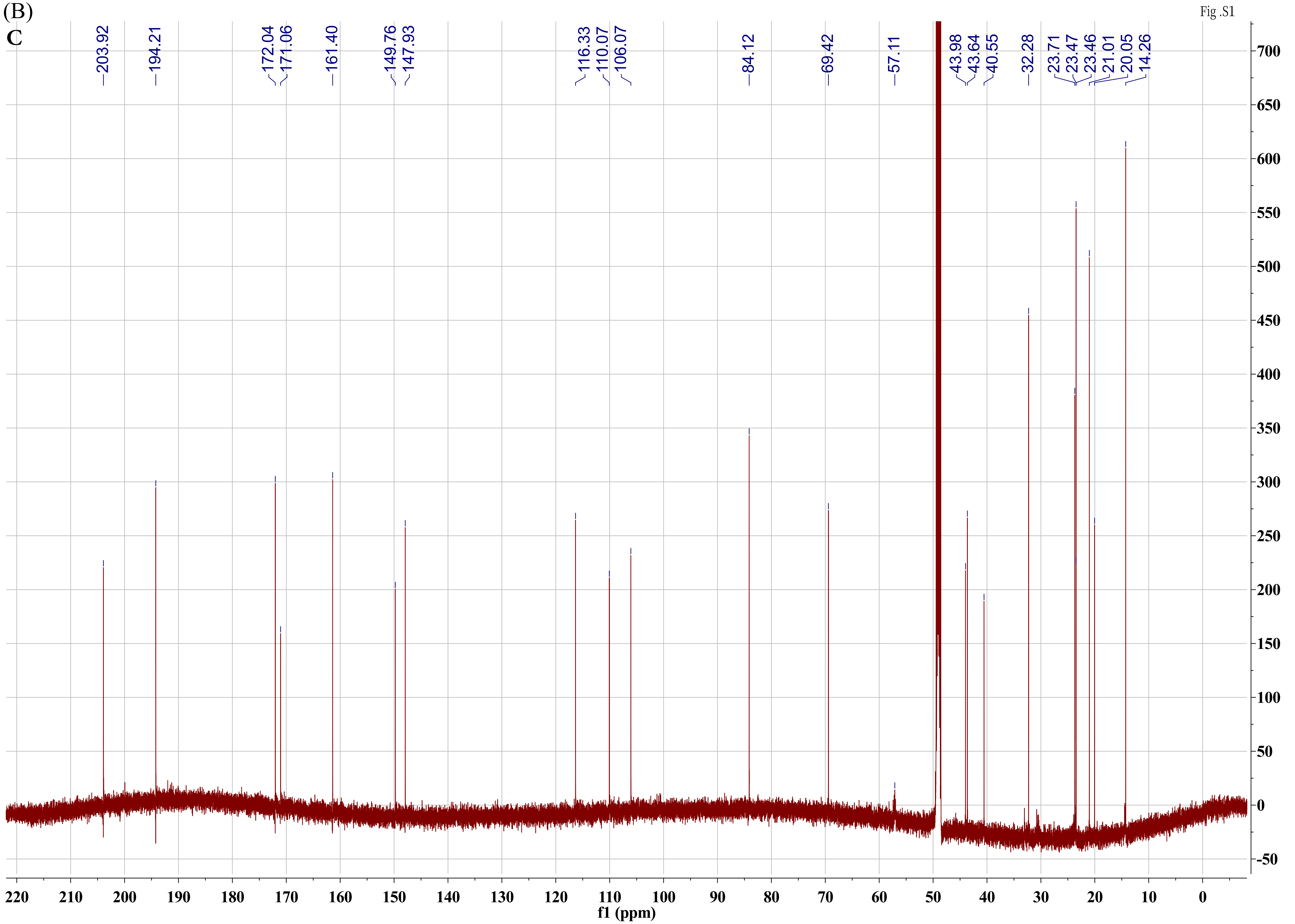
Fig .S1
C
203.92
194.21
172.04
171.06
161.40
149.76
147.93
116.33
110.07
106.07
84.12
69.42
57.11
43.98
43.64
40.55
32.28
23.71
23.47
23.46
21.01
20.05
14.26
f1 (ppm)

(C)

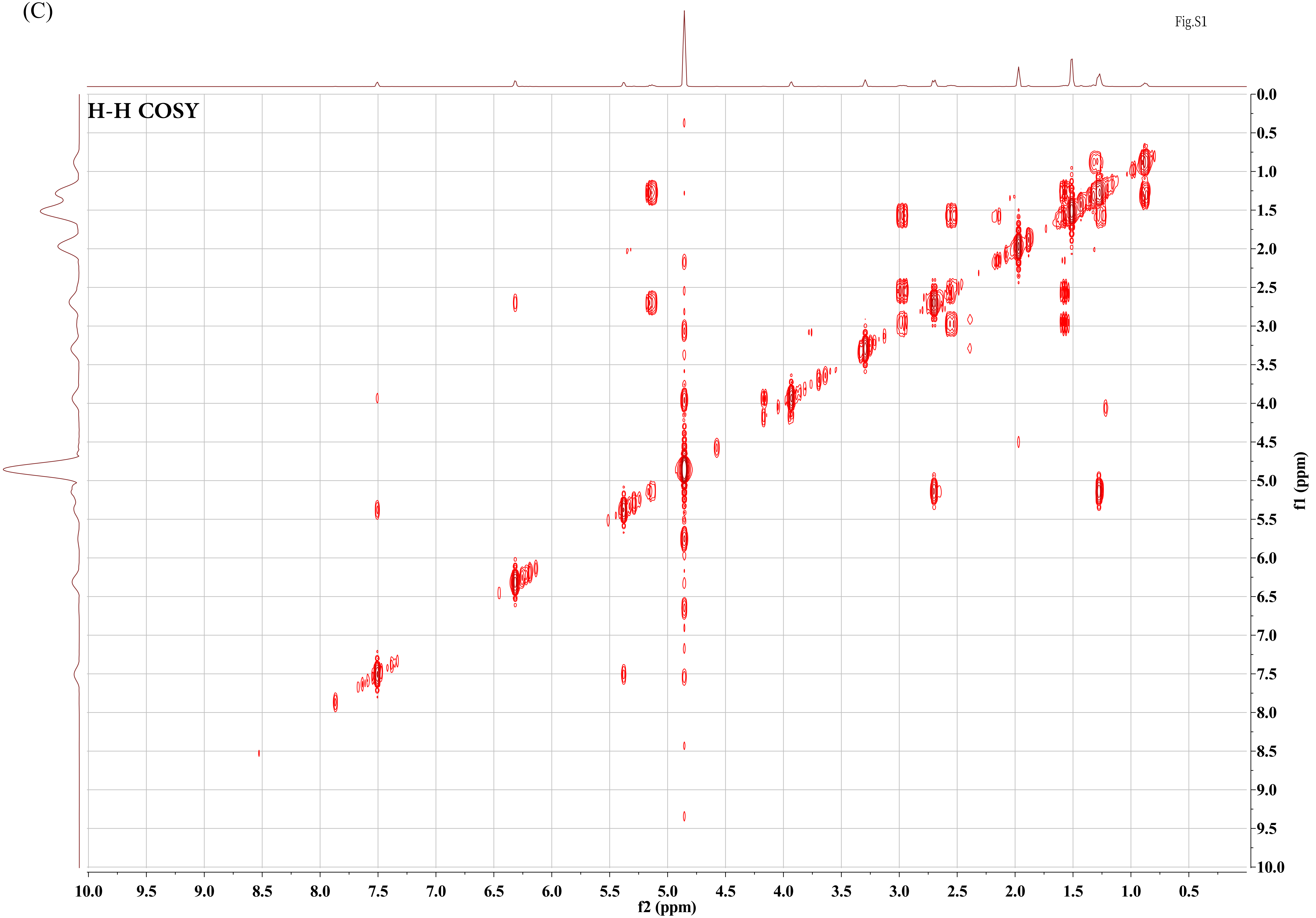
Fig.S1
H-H COSY
f2 (ppm)
f1 (ppm)
10.0
9.5
9.0
8.5
8.0
7.5
7.0
6.5
6.0
5.5
5.0
4.5
4.0
3.5
3.0
2.5
2.0
1.5
1.0
0.5
0.0

(D)

Fig.S1

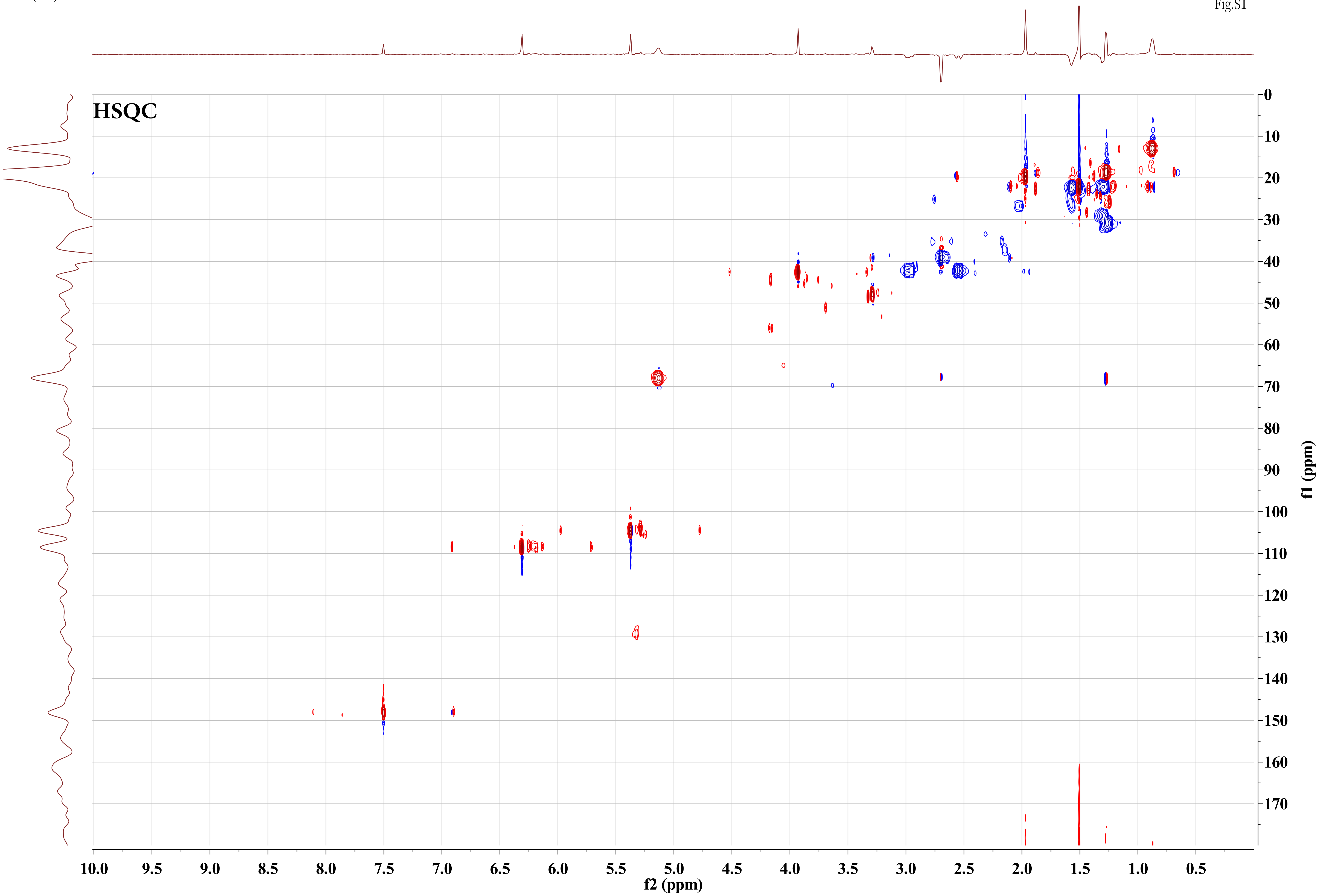
HSQC
10.0
9.5
9.0
8.5
8.0
7.5
7.0
6.5
6.0
5.5
5.0
4.5
4.0
3.5
3.0
2.5
2.0
1.5
1.0
0.5
f2 (ppm)
0
10
20
30
40
50
60
70
80
90
100
110
120
130
140
150
160
170
f1 (ppm)

(E)

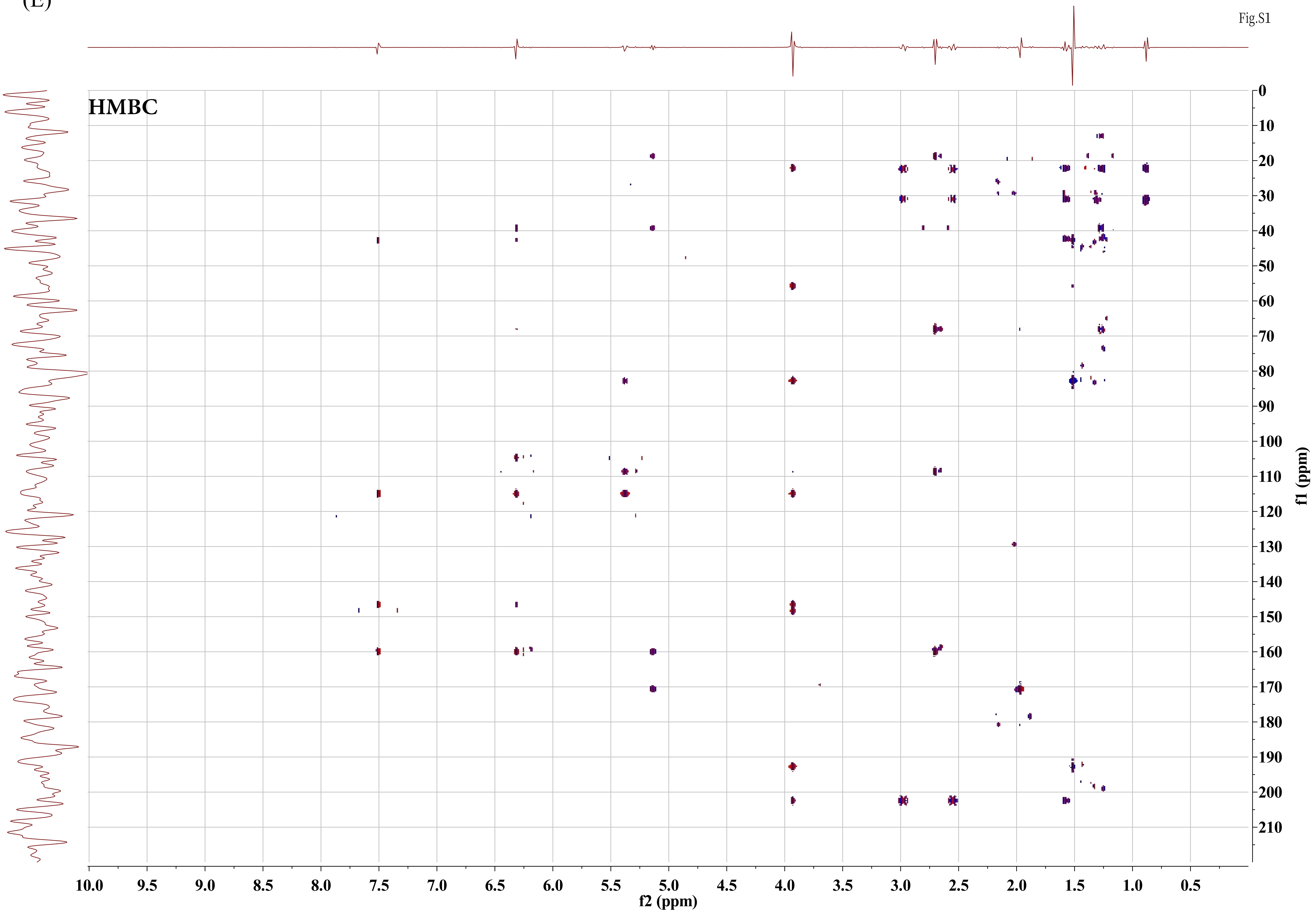

(A)

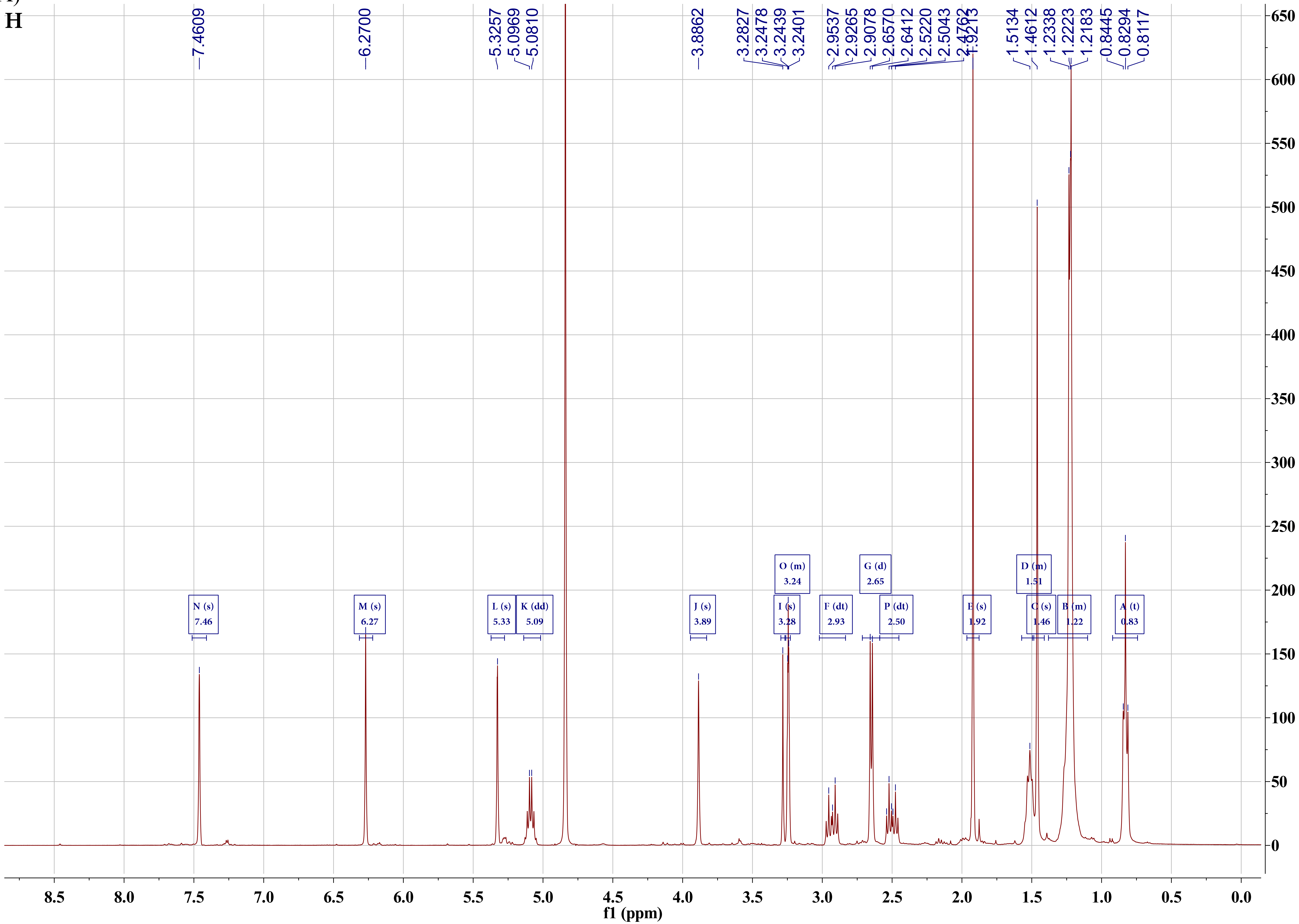
H
7.4609
6.2700
5.3257
5.0969
5.0810
3.8862
3.2827
3.2478
3.2439
3.2401
2.9537
2.9265
2.9078
2.6570
2.6412
2.5220
2.5043
2.4762
1.9213
1.5134
1.4612
1.2338
1.2223
1.2183
0.8445
0.8294
0.8117
N (s) 7.46
M (s) 6.27
L (s) 5.33
K (dd) 5.09
J (s) 3.89
O (m) 3.24
I (s) 3.28
F (dt) 2.93
G (d) 2.65
P (dt) 2.50
E (s) 1.92
D (m) 1.51
C (s) 1.46
B (m) 1.22
A (t) 0.83
f1 (ppm)

(B)

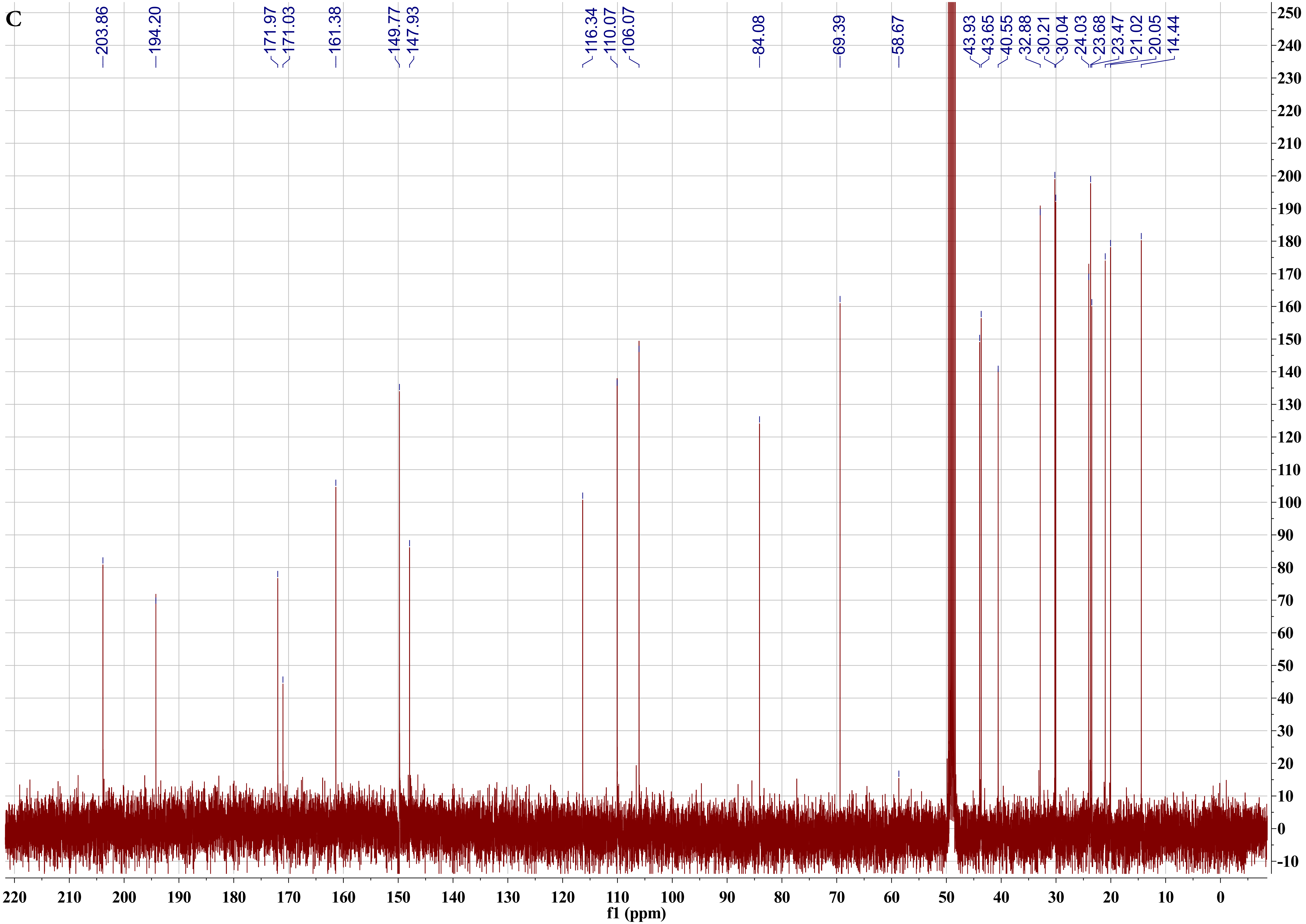
C
203.86
194.20
171.97
171.03
161.38
149.77
147.93
116.34
110.07
106.07
84.08
69.39
58.67
43.93
43.65
40.55
32.88
30.21
30.04
24.03
23.68
23.47
21.02
20.05
14.44
f1 (ppm)

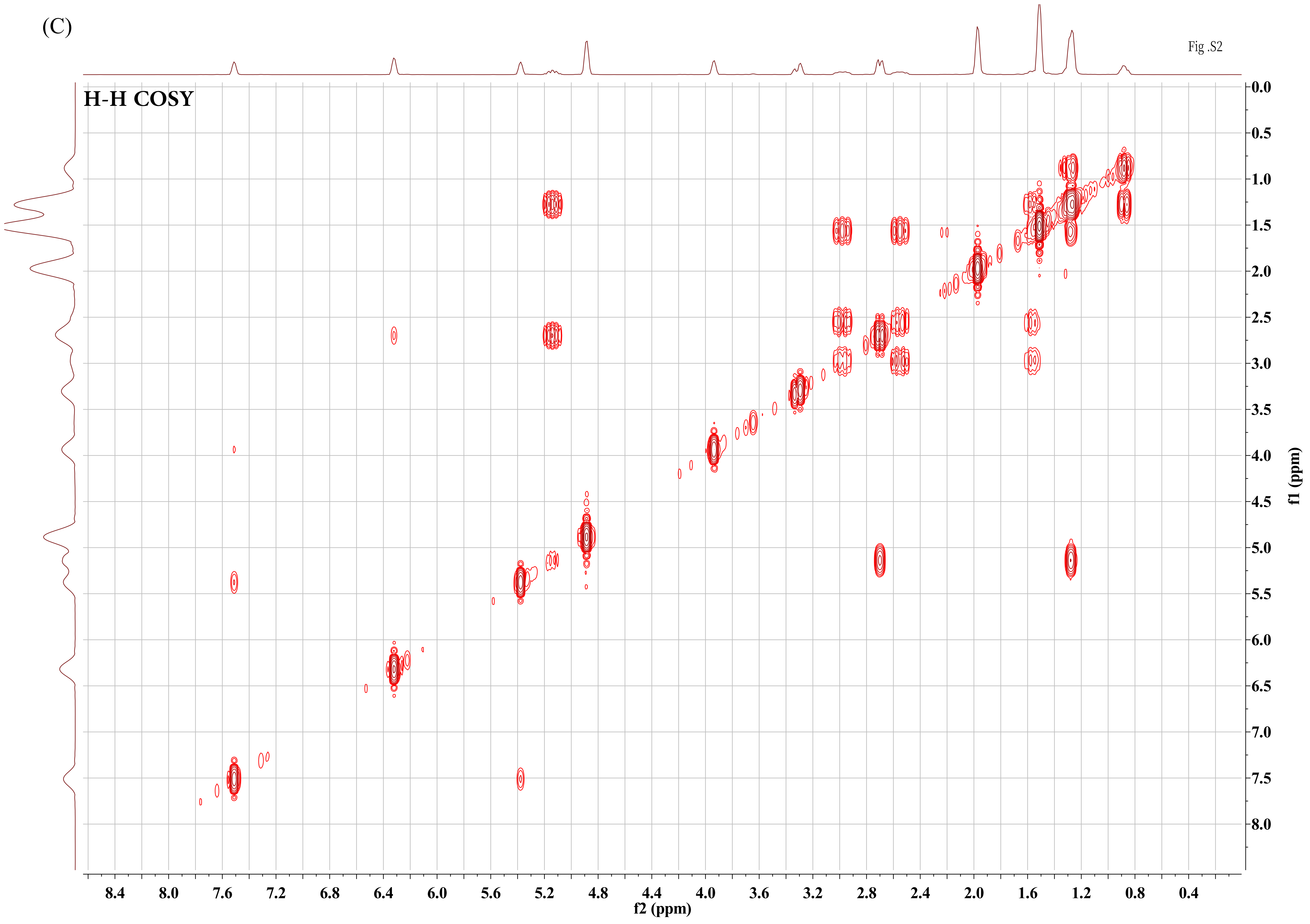
(C)
Fig .S2
H-H COSY
8.4
8.0
7.6
7.2
6.8
6.4
6.0
5.6
5.2
4.8
4.4
4.0
3.6
3.2
2.8
2.4
2.0
1.6
1.2
0.8
0.4
f2 (ppm)
0.0
0.5
1.0
1.5
2.0
2.5
3.0
3.5
4.0
4.5
5.0
5.5
6.0
6.5
7.0
7.5
8.0
f1 (ppm)

(D)

Fig.S2

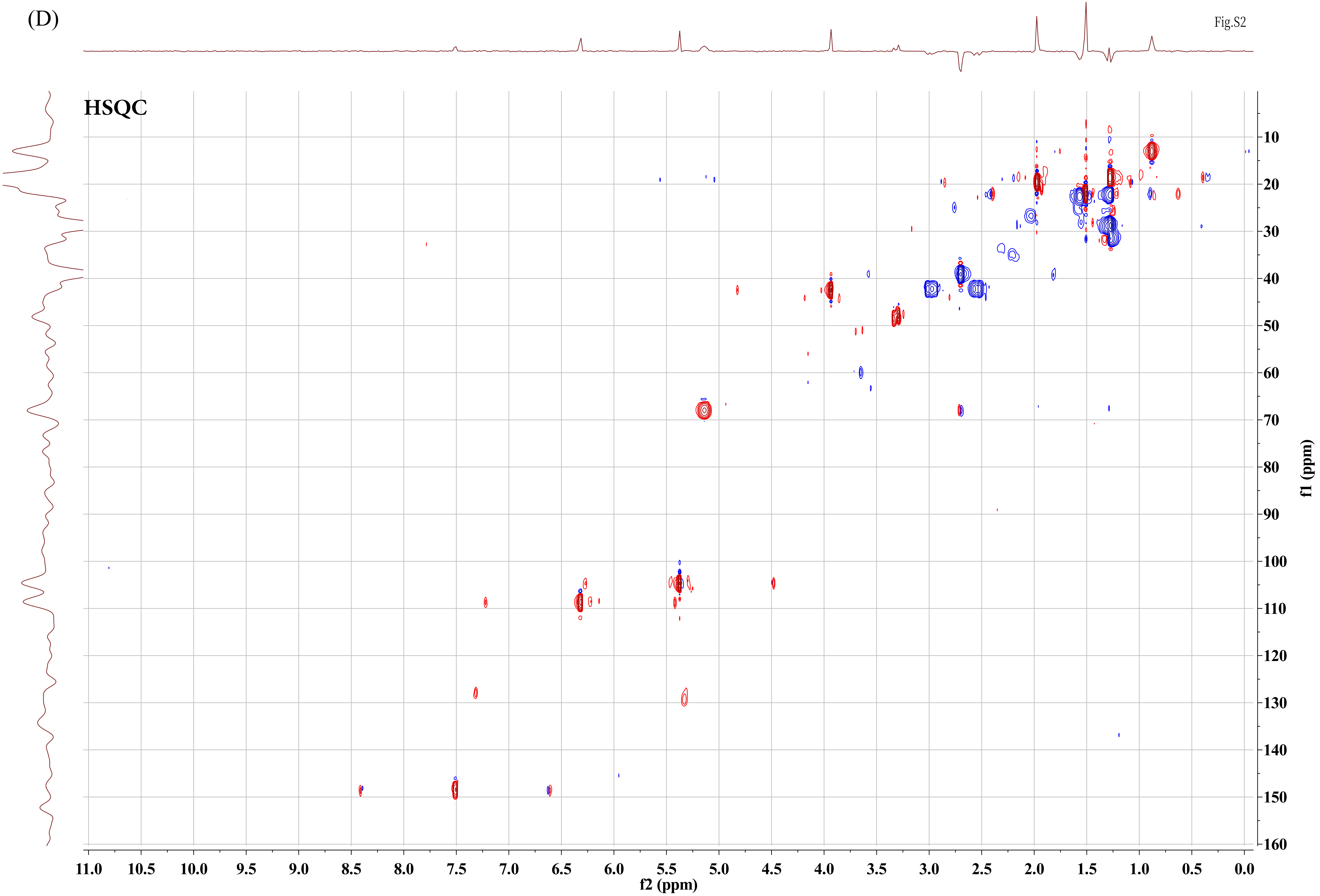

HSQC
f1 (ppm)
f2 (ppm)
11.0 10.5 10.0 9.5 9.0 8.5 8.0 7.5 7.0 6.5 6.0 5.5 5.0 4.5 4.0 3.5 3.0 2.5 2.0 1.5 1.0 0.5 0.0
10 20 30 40 50 60 70 80 90 100 110 120 130 140 150 160

(E)

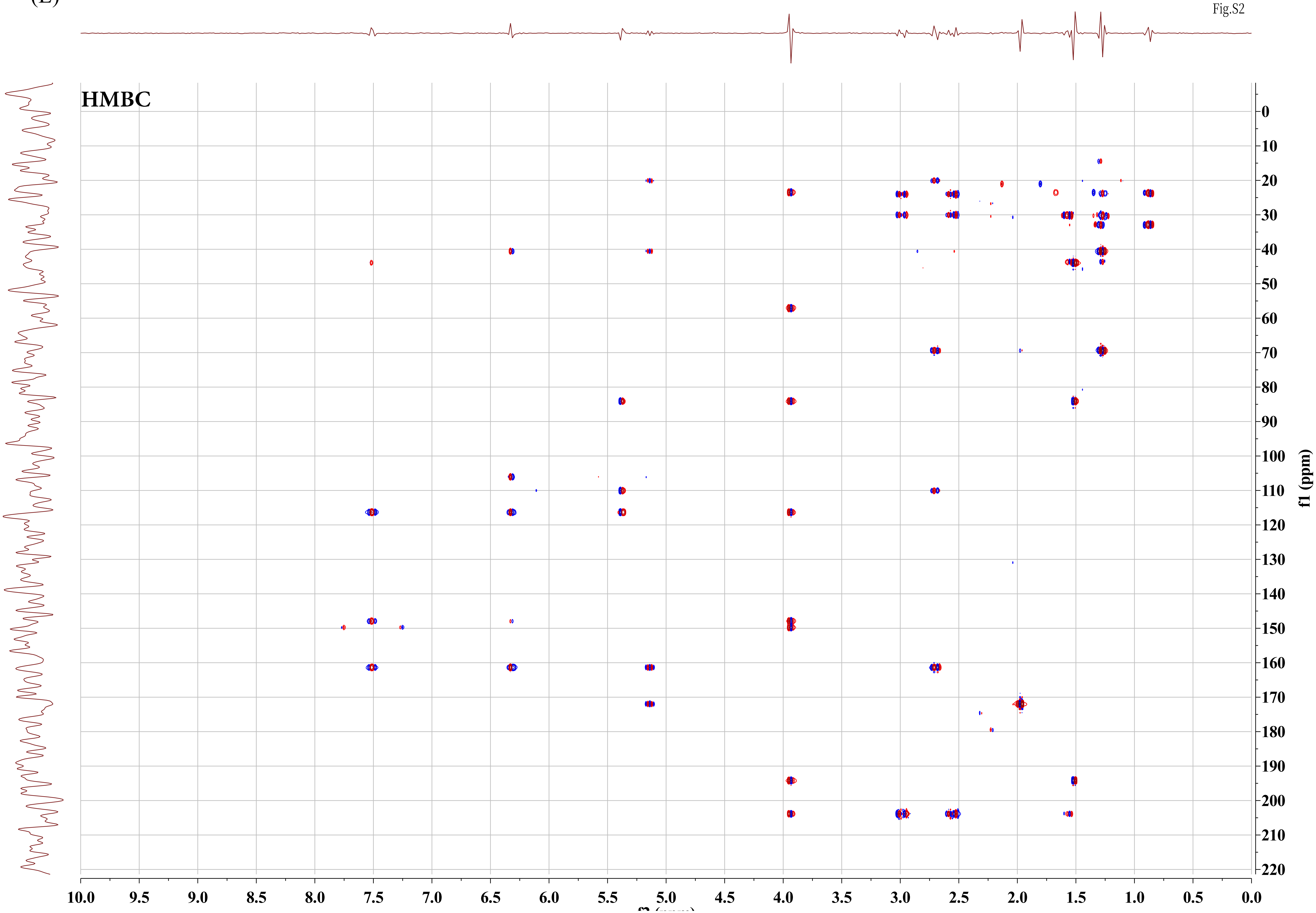

Supplement: Supplementary file 1 — Additional file 1: Figure S1. NMR spectra for compound 2. (A) H-NMR. (B) C-NMR. (C) COSY. (D) HMBC. (E) HSQC. (F)TOCSY. Figure S2. NMR spectra for compound 3. (A) H-NMR. (B) C-NMR. (C) COSY. (D) HMBC. (E) HSQC. (F)TOCSY. [file 13568_2017_467_MOESM1_ESM.pdf]
